# Morphometric and radiomics analysis toward the prediction of epilepsy associated with supratentorial low-grade glioma in children

**DOI:** 10.1186/s40644-025-00881-1

**Published:** 2025-05-19

**Authors:** Min-Lan Tsai, Kevin Li-Chun Hsieh, Yen-Lin Liu, Yi-Shan Yang, Hsi Chang, Tai-Tong Wong, Syu-Jyun Peng

**Affiliations:** 1 Department of Pediatrics, Taipei Medical University Hospital, Taipei Medical University, Taipei, Taiwan; 2 Department of Pediatrics, School of Medicine, College of Medicine, Taipei Medical University, Taipei, Taiwan; 3 Department of Medical Imaging, School of Medicine, Taipei Medical University Hospital, Taipei, Taiwan; 4 Department of Radiology, School of Medicine, College of Medicine, Taipei Medical University, Taipei, Taiwan; 5 Division of Pediatric Neurosurgery, Department of Neurosurgery, Taipei Medical University Hospital, Taipei, Taiwan; 6 Institute of Clinical Medicine, College of Medicine, Taipei Medical University, Taipei, Taiwan; 7 Taipei Neuroscience Institute, Taipei Medical University Hospital, Taipei, Taiwan; 8 In-Service Master Program in Artificial Intelligence in Medicine, College of Medicine, Taipei Medical University, No.250, Wuxing St., Xinyi Dist, Taipei City, 110 Taiwan; 9 Clinical Big Data Research Center, Taipei Medical University Hospital, Taipei Medical University, Taipei, Taiwan

**Keywords:** Radiomics, Supratentorial glioma, Low-grade glioma, Children, Glioma-related epilepsies, T2-flair image

## Abstract

**Objectives:**

Understanding the impact of epilepsy on pediatric brain tumors is crucial to diagnostic precision and optimal treatment selection. This study investigated MRI radiomics features, tumor location, voxel-based morphometry (VBM) for gray matter density, and tumor volumetry to differentiate between children with low grade glioma (LGG)-associated epilepsies and those without, and further identified key radiomics features for predicting of epilepsy risk in children with supratentorial LGG to construct an epilepsy prediction model.

**Methods:**

A total of 206 radiomics features of tumors and voxel-based morphometric analysis of tumor location features were extracted from T2-FLAIR images in a primary cohort of 48 children with LGG with epilepsy (*N* = 23) or without epilepsy (*N* = 25), prior to surgery. Feature selection was performed using the minimum redundancy maximum relevance algorithm, and leave-one-out cross-validation was applied to assess the predictive performance of radiomics and tumor location signatures in differentiating epilepsy-associated LGG from non-epilepsy cases.

**Results:**

Voxel-based morphometric analysis showed significant positive t-scores within bilateral temporal cortex and negative t-scores in basal ganglia between epilepsy and non-epilepsy groups. Eight radiomics features were identified as significant predictors of epilepsy in LGG, encompassing characteristics of 2 locations, 2 shapes, 1 image gray scale intensity, and 3 textures. The most important predictor was temporal lobe involvement, followed by high dependence high grey level emphasis, elongation, area density, information correlation 1, midbrain and intensity range. The Linear Support Vector Machine (SVM) model yielded the best prediction performance, when implemented with a combination of radiomics features and tumor location features, as evidenced by the following metrics: precision (0.955), recall (0.913), specificity (0.960), accuracy (0.938), F-1 score (0.933), and area under curve (AUC) (0.950).

**Conclusion:**

Our findings demonstrated the efficacy of machine learning models based on radiomics features and voxel-based anatomical locations in predicting the risk of epilepsy in supratentorial LGG. This model provides a highly accurate tool for distinguishing epilepsy-associated LGG in children, supporting precise treatment planning.

**Trial registration:**

Not applicable.

**Supplementary Information:**

The online version contains supplementary material available at 10.1186/s40644-025-00881-1.

## Introduction

Epilepsy can significantly impact the quality of life and morbidity of children with brain tumors [1]. It has been estimated that approximately 70% of pediatric patients with LGGs experience seizures during the disease course, and 30–50% of pediatric patients with LGGs have seizures on presentation [2, 3, 4]. Patients with low grade gliomas (LGG) are more susceptible to seizures than are those with high-grade gliomas [5]. Previous cohort studies have demonstrated that tumor location and pathology are major risk factors for development of epilepsy; however, this relationship has yet to be fully elucidated.

The pathogenesis of glioma related epilepsy was associated with multiple mechanisms involving disruption of blood-brain barrier, molecular changes, edema, and peritumoral environmental changes [1, 4]. Factors affecting epileptogenesis include tumor location, growth rate, altered neurotransmitter homeostasis, molecular alterations, homoeostasis between astrocytes and synaptic transmission, and gap junction alterations [6, 7].

Radiomics is an emerging technique that utilizes quantitative imaging features, such as those obtained from MRI, to guide clinical diagnosis, treatment decisions, and prognosis evaluations [8]. Many of these features are not immediately evident to clinicians; however, when integrated with other relevant data sources, they can have a profound effect on the accuracy of predictive and diagnostic models. Researchers have recently proposed combining MRI-based radiomics with quantitative volumetric or spatial mapping analysis of tumor displacement to assess epileptogenic potential [9, 10].

MRI radiomics has proven useful in differentiating between high-grade and low-grade tumors, as well as in predicting the pathology of brain tumors [11]. In adult patients, MRI radiomics has also proven useful in differentiating between brain tumors associated with epilepsy from those without [12, 13]. Our study focuses on predicting epileptic seizures in the pediatric population with supratentorial LGGs, which has not been studied as extensively as in the adult population.

This study investigated MRI radiomics features, tumor location, voxel-based morphometry (VBM) for gray matter density, and tumor volumetry to differentiate between children with LGG-associated epilepsies and those without. Moreover, we developed a feature screening algorithm and validated a radiomics-based model designed to predict the occurrence of epilepsy in children with LGG. We hypothesize that our model, integrating radiomics features and morphologic characteristics, can accurately predict tumor-associated epilepsies. This predictive capability could enable early intervention for high-risk patients, while preventing unnecessary treatment for low-risk patients.

## Patients and methods

### Clinical demographics

This retrospective study identified 229 brain tumor patients in the database of the Pediatric Brain Tumor Program, Taipei Medical University Hospital for the period between January 2014 and December 2023. Inclusion criteria included a diagnosis of a primary brain tumor at 18 years or younger with complete MRI survey. Based on initial histology, high-grade and infratentorial tumors were excluded, leaving only cases involving low-grade gliomas. Exclusion criteria included the initiation of antitumoral therapy prior to preoperative MRI scans and other lesions that could cause epilepsy, such as cerebral hemorrhage, stroke, or other brain tumors. After applying these criteria, 48 children with supratentorial LGG (WHO grades I-II) were included in the study. Among these, 23 patients with two or more habitual seizures were classified in the epilepsy group, while the other 25 patients were classified in the non- epilepsy group. Our two groups were balanced in terms of class distribution (48% vs. 52%).

Medical records were reviewed to extract the following clinical data: age, gender, tumor site, tumor pathology, seizure type, seizure frequency, major symptoms, electroencephalography (EEG) or video EEG findings, anti-seizure medications, and seizure outcomes. All patients underwent surgical resection or biopsy with complete MRI survey. Tumor location and 3D features were assessed prior to surgical intervention. This study was approved by the ethics committee of the Institutional Review Board (IRB) of Taipei Medical University Hospital (TMU-JIRB-N202405060).

### MRI analysis

Preoperative MRI examinations were conducted using T2-weighted-Fluid-Attenuated Inversion Recovery (T2-FLAIR) images captured using a 3-T scanner with a 20-channel head coil (Siemens MAGNETOM Prisma, Erlangen, Germany). Axial T2-FLAIR images were acquired using the following parameters: pixel matrix (256 × 208), field of view (240 × 195 mm), inversion time (2371.9 ms), echo time (83 ms), repetition time (8000 ms), and section thickness (4 mm without a gap). MRI scans of poor quality due to head motion or misalignment artifacts were excluded.

This study sought to differentiate between epilepsy and non- epilepsy groups based on tumor location and tumor characteristics, including cystic content, peritumor edema, the presence of hydrocephalus, multiple lobe involvement, 3D tumor volume, and involvement of the basal ganglia, thalamus, or midbrain.

### Radiomics analysis

**Fig. 1:**
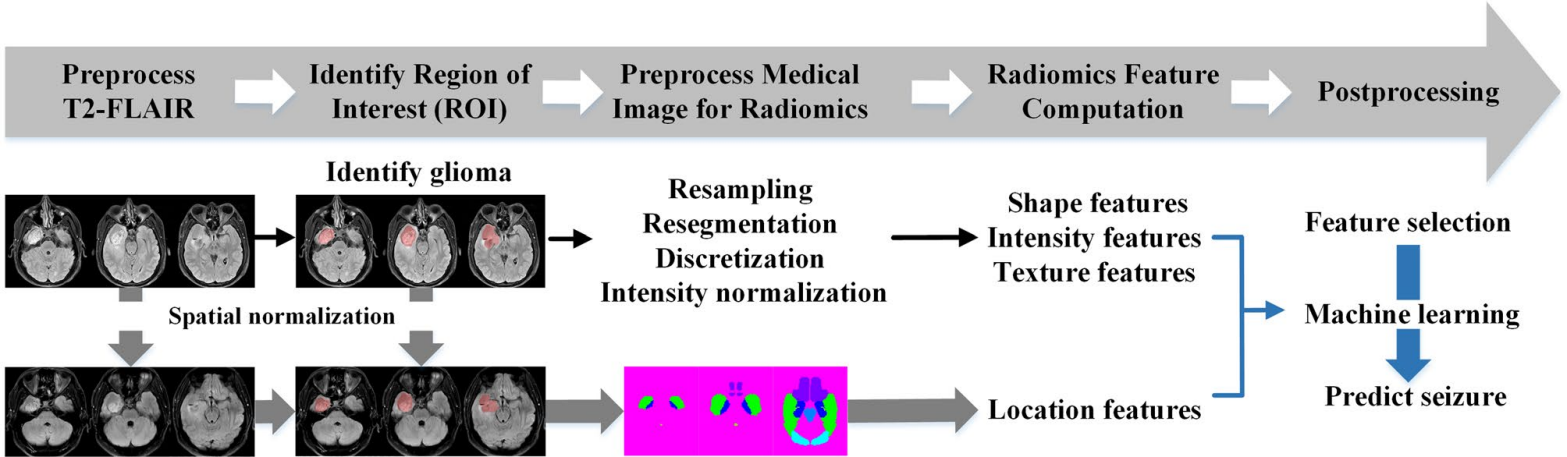
Flow chart illustrating the process of image preparation, computation, and seizure prediction. After manual delineation of tumors in T2 flair images to identify the Region of interest (ROI), spatial normalization was performed, and tumor location features were extracted. Radiomics feature extraction was performed after resampling, resegmentation, discretization, and intensity normalization. Radiomics features were analyzed in terms of shape, intensity, texture, and location. Key radiomics and location features were integrated into a machine learning model for seizure prediction

Figure 1. illustrates the computation and prediction process employed in this study. Tumor regions of interest (ROIs) were manually delineated by two experienced neurologists (Tsai and Chang) with mutual agreement. To ensure consistency, all delineated ROIs were subsequently reviewed and finalized by mutual consensus. Feature extraction from tumor ROIs was performed using the radiomics package in the MATLAB R2023a Medical Image Toolbox (MathWorks, Natick, MA, USA). Preprocessing steps included resampling, resegmentation, discretization, and intensity normalization [14, 15]. All images were resampled to an isotropic voxel spacing of 1 × 1 × 1 mm³. Intensity normalization was performed using z-score normalization within the tumor region of interest (ROI). For intensity discretization, a fixed bin width of 25 Gy levels was applied. Segmentation and all preprocessing steps, including resegmentation and image filtering, were conducted in accordance with the Image Biomarker Standardization Initiative (IBSI) guidelines.

A total of 206 radiomics features from the T2-FLAIR images were categorized as follows: shape (21), intensity (49), texture (136), and tumor location (10). Tumor locations were categorized according to the WFU_PickAtlas (https://www.nitrc.org/projects/wfu_pickatlas/), as follows: frontal lobe, limbic lobe, occipital lobe, parietal lobe, temporal lobe, sublobar regions (excluding the insula, caudate, putamen, pallidum, and thalamus), insula, basal ganglia (caudate, putamen, and pallidum), thalamus, and midbrain.

The relationship between clinical and radiologic characteristics was also examined. After feature selection, we developed radiomics prediction models based on machine learning techniques, including decision trees, discriminant analysis, logistic regression classifiers, naïve bayes classifiers, support vector machines, and nearest neighbor classifiers.

### Voxel-based analysis of tumor location

Voxel-based tumor location analysis was conducted in MATLAB (MathWorks, Inc., Natick, MA, USA) using Statistical Parametric Mapping software (SPM12) (Functional Imaging Laboratory, Institute of Neurology, University College London, London, UK).

 [16, 17]. To facilitate normalization to the standard brain space, the origin of the T2-FLAIR image was adjusted to align approximately with the anterior commissure of the individual brain space. Each T2-FLAIR image was then spatially aligned to the MNI152 template in the Montreal Neurological Institute (MNI) space using the SPM12 normalization tool, which computed a transformation matrix. The matrix was subsequently applied to normalize the tumor location image to the standard MNI space.

Following normalization, the tumor location image underwent spatial smoothing using a spatially stationary Gaussian filter. To enhance the signal-to-noise ratio for between-group statistical analysis, a Gaussian smoothing kernel with a full width at half maximum (FWHM) of 4 mm was applied. The spatial smoothing kernel (FWHM = 4 mm) was selected to balance sensitivity and spatial specificity in pediatric brain imaging. Given the relatively small brain volume and high anatomical variability in children, a moderate smoothing kernel helps improve the signal-to-noise ratio without excessively blurring fine anatomical structures. This kernel size has been commonly applied in pediatric neuroimaging studies involving gray matter density analysis [18, 19]. The minimum cluster size threshold of 54 voxels was determined using AlphaSim correction, which estimates the probability of false-positive clusters based on Monte Carlo simulations. Our threshold corresponds to a corrected *p*-value < 0.05, accounting for multiple comparisons across the brain volume. Simulations were performed using the parameters of our normalized voxel size, image dimensions, and the applied 4 mm smoothing kernel. The null hypothesis stated that there was no difference in tissue volume between epilepsy and non- epilepsy groups. These results were visualized as color maps, with the t-statistic represented by the scale. *P*-values were adjusted for multiple comparisons, and age and gender were included as a covariate in the analysis.

### Feature screening and validation for prediction model

Feature screening was conducted using the rank features for classification method with the minimum redundancy maximum relevance (mRMR) algorithm, a technique widely used for feature selection in classification tasks [20]. The mRMR algorithm identifies features that are most discriminative for target variables, such as classification labels, while minimizing redundancy among features.

Prediction performance was assessed using leave-one-out cross-validation focusing on radiomics and tumor location features both separately and in combination. After prediction validation, the optimal model was evaluated for prediction of epilepsy, leveraging radiomics features and/or tumor location, following prediction validation.

The receiver operating characteristic curve (ROC) and area under curve (AUC) were used to evaluate the classification performance of the models in each cohort.

### Statistical analysis

Demographic variables were reported as counts and percentages for analysis using either the chi-square test or Fisher’s exact test, depending on whether the expected cell count was less than 5. Between-group comparisons of continuous variables were conducted using the Mann-Whitney U test or unpaired t-tests, as appropriate. Statistical analysis was performed using the Statistics and Machine Learning Toolbox in the MATLAB R2023a environment. A two-sided *p*-value of less than 0.05 was considered statistically significant for all hypothesis tests.

Differences in the normalized tumor location images between epilepsy and non- epilepsy groups were analyzed using two-sample t-tests with gender and age as covariates. A dual statistical threshold was applied, consisting of a height threshold of *p* < 0.01 and a minimum cluster size of 54 voxels, both of which were determined using AlphaSim correction.

## Results

### Clinical characteristics

A total of 48 children with low grade glioma were included in this study. Table 1 lists the clinical parameters, including tumor grade, age at the time of diagnosis, age at the time of seizure onset, gender, seizure frequency and duration, seizure type and anti-seizure medication (if applicable). No significant difference was detected between the groups in terms of age, gender, or tumor grade.

**Table 1:** Clinical demographics of pediatric supratentorial low grade glioma patients with or without epilepsy

Variables	with epilepsy (no. of patients)	without epilepsy (no. of patients)	*p* value
Patient number	23	25	
Gender (male %)	15 (65.2%)	10 (40%)	0.15
Age at diagnosis (MRI) (years old, mean ± SD)	11.3 ± 6.3 years (range 1.0–18)	10.6 ± 5.8 years (range 1.0–18)	0.76
Age of seizure onset (years old, mean ± SD)	10.6 ± 7.8 years		
Seizure frequency (monthly)	17 (73.9%)		
Seizure duration (mins)	4.2 ± 5.3		
Focal onset seizure	18 (78.2%)		
Focal to FBTCS	5 (21.7%)		
ASM polytherapy	11 (47.8%)		
**MRI characteristics**			
Side (right %)	8 (34.7%)	15 (60%)	0.15
Temporal lobe (main mass)	17 (73.9%)	4 (16%)	0.000088**
Basal ganglia involvement	6 (26.0%)	17 (68%)	0.008932**
Midbrain involvement	2 (8.6%)	10 (40%)	0.03*
Multiple lobes	6 (26.1%)	19 (76%)	0.0015**
Cyst formation	14 (60.9%)	8 (32%)	0.08626
Peritumor edema	11 (47.8%)	7 (28%)	0.26314
Hydrocephalus at diagnosis	1 (4.3%)	1 (4%)	0.50752
Tumor volume (ml)	34.61 ± 35.63	19.79 ± 21.57	0.085
**Tumor pathology**			
Grade 1	13 (56.5%)	14 (56%)	0.79887
Grade 2	10 (43.4%)	11 (44%)	0.79887

ASM: anti-seizure medication; FBTCS: focal to bilateral tonic-clonic seizures; SD: standard deviation; Unpaired T-test or nonparametric Mann-Whitney (M-W) two-sample test / Chi-square test with Yate’s correction/Fisher exact test where appropriate. * *p* < 0.05; ** *p* < 0.01

Based on the visual interpretation of MRI scans, it was determined that tumor location in the temporal lobe was significantly more common in the epilepsy group (73.9%) compared to the non- epilepsy group (16%) (*p* < 0.001). Basal ganglia involvement was significantly higher in the non-epilepsy group (68%), compared to the epilepsy group (26%) (*p* = 0.009). Midbrain involvement was significantly higher in the non-epilepsy group (40%), compared to the epilepsy group (8.6%) (*p* = 0.03). The risk of multiple lobe involvement was significantly lower in the epilepsy group (26.1%), than in the non- epilepsy group (76%) (*p* = 0.0015). No significant differences were detected between the epilepsy and non- epilepsy groups in terms of cyst formation, peritumor edema, tumor volume, or the presence of hydrocephalus.

### Voxel-based analysis of tumor location

**Fig. 2:**
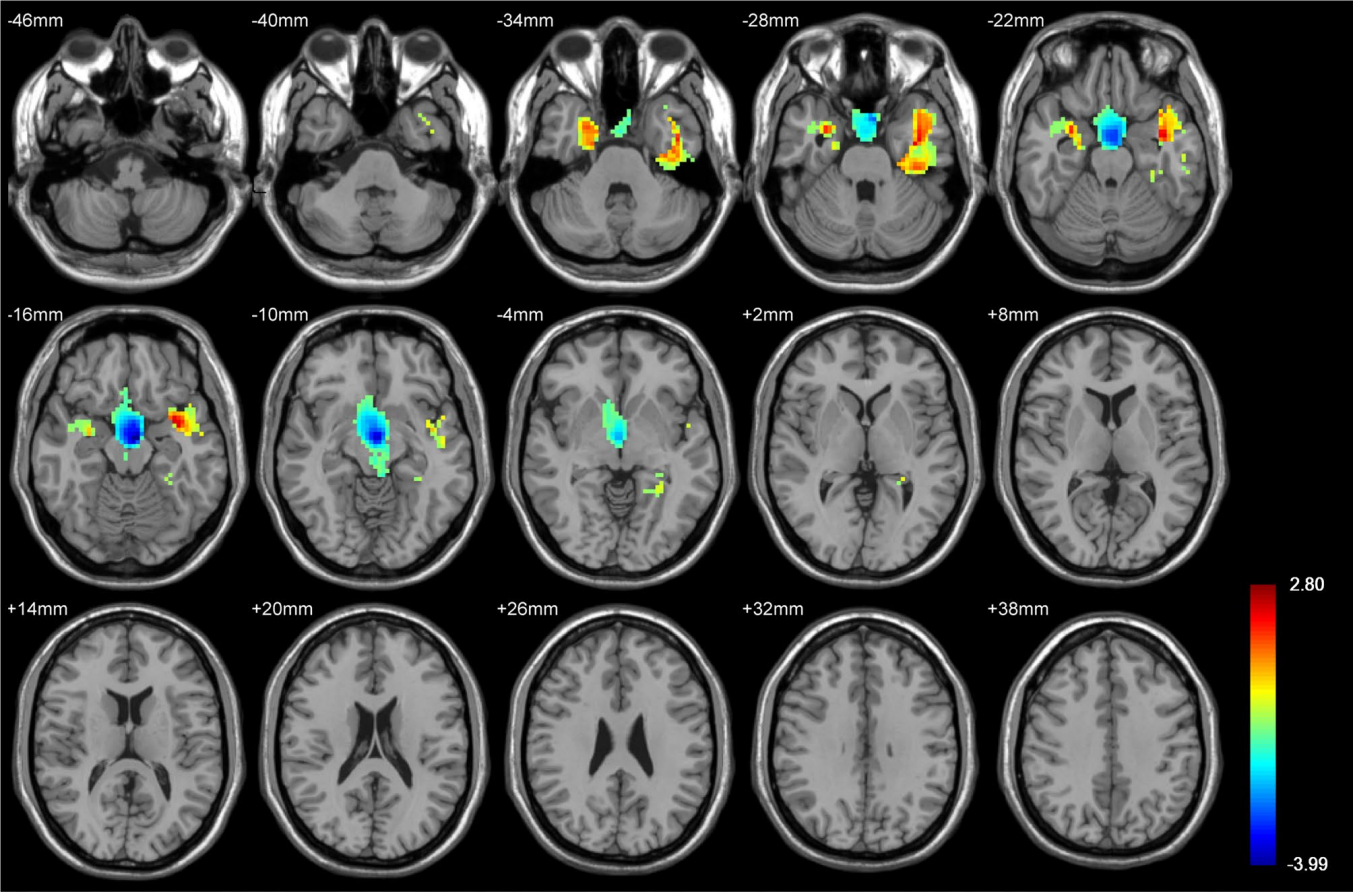
Voxel-based analysis showing differences in tumor location between epilepsy and non- epilepsy groups. The color bar represents t-scores, where red indicates higher t-scores and blue indicates negative t-scores. The epilepsy group presented higher positive t-scores in the temporal region (red and yellow) and negative t-scores in the basal ganglia and midbrain region (blue). Differences with a *p* < 0.01 were considered statistically significant. The peak MNI coordinates of the significant clusters identified in the voxel-based analysis. Specifically, the epilepsy group showed significantly increased tumor involvement in the temporal cortex with peak MNI coordinates at (-36, 0, -18) and (30, 0, -24), while the non-epilepsy group demonstrated significant involvement in the basal ganglia and midbrain with a peak MNI coordinate at (-3, -9, -12)

Voxel-based analysis was performed to compare tumor locations between the epilepsy and non- epilepsy groups. T-scores derived from a two-sample t-test accounted for multiple comparisons, with age and gender included as covariates. As shown in Fig. 2, positive t-scores in tumor locations within the bilateral temporal cortex were significantly higher in the epilepsy group than in the non- epilepsy group. Conversely, the non- epilepsy group exhibited negative t-scores for the basal ganglia and parts of the midbrain, indicating significant gray matter involvement in these regions.

### Radiomics analysis

#### Predicting risk of epilepsy: tumor location

As shown in Table 2, the cubic Support Vector Machines (SVM) yielded the best prediction performance, based on tumor location features and voxel-based morphometry (VBM) features, as evidenced by the following metrics: Precision (0.760), recall (0.826), specificity (0.760), accuracy (0.792), F-1 score (0.792), and AUC (0.870).

**Table 2:** Performance of seizure prediction models using tumor location features for pediatric patients with supratentorial glioma

Classifier	Precision	Recall	Specificity	Accuracy	F1-Score	TP	FN	FP	TN	AUC
Cubic SVM	0.760	0.826	0.760	0.792	0.792	19	4	6	19	0.870
Weighted KNN	0.783	0.783	0.800	0.792	0.783	18	5	5	20	0.835
Fine KNN	0.826	0.826	0.840	0.833	0.826	19	4	4	21	0.833
Cosine KNN	0.714	0.870	0.680	0.771	0.784	20	3	8	17	0.814
Linear Discriminant	0.923	0.522	0.960	0.750	0.667	12	11	1	24	0.807
Binary GLM Logistic Regression	0.857	0.522	0.920	0.729	0.649	12	11	2	23	0.803
Medium KNN	0.714	0.870	0.680	0.771	0.784	20	3	8	17	0.800
Cubic KNN	0.714	0.870	0.680	0.771	0.784	20	3	8	17	0.794
Fine Tree	0.741	0.870	0.720	0.792	0.800	20	3	7	18	0.788
Medium Tree	0.741	0.870	0.720	0.792	0.800	20	3	7	18	0.788
Coarse Tree	0.741	0.870	0.720	0.792	0.800	20	3	7	18	0.788
Fine Gaussian SVM	0.750	0.522	0.840	0.688	0.615	12	11	4	21	0.779
Medium Gaussian SVM	0.800	0.522	0.880	0.708	0.632	12	11	3	22	0.767
Gaussian Naive Bayes	0.923	0.522	0.960	0.750	0.667	12	11	1	24	0.760
Coarse Gaussian SVM	0.706	0.522	0.800	0.667	0.600	12	11	5	20	0.760
Quadratic Discriminant	0.917	0.478	0.960	0.729	0.629	11	12	1	24	0.757
Quadratic SVM	0.690	0.870	0.640	0.750	0.769	20	3	9	16	0.708
Linear SVM	0.818	0.391	0.920	0.667	0.529	9	14	2	23	0.694
Kernel Naive Bayes	0.875	0.609	0.920	0.771	0.718	14	9	2	23	0.692
Coarse KNN	NaN	0.000	1.000	0.521	NaN	0	23	0	25	0.000

AUC, area under receiver operating characteristic curve; FN: False Negative; FP: False Positive; GLM: Generalized linear models; KNN: k-nearest neighbors; SVM: Support Vector Machines; TN: True Negative TP: True Positive

#### Predicting risk of epilepsy: tumor radiomics

As shown in Table 3, the Coarse Gaussian Support Vector Machine (SVM) model yielded the best prediction performance, based on radiomics features related to shape, intensity, and texture, as evidenced by the following metrics: Precision (0.800), recall (0.870), specificity (0.800), accuracy (0.833), F-1 score (0.833), and AUC (0.859).

**Table 3:** Performance of seizure prediction models using radiomics features for pediatric patients with supratentorial glioma

Classifier	Precision	Recall	Specificity	Accuracy	F1-Score	TP	FN	FP	TN	AUC
Coarse Gaussian SVM	0.800	0.870	0.800	0.833	0.833	20	3	5	20	0.859
Medium KNN	0.850	0.739	0.880	0.813	0.791	17	6	3	22	0.851
Linear Discriminant	0.800	0.870	0.800	0.833	0.833	20	3	5	20	0.845
Linear SVM	0.800	0.870	0.800	0.833	0.833	20	3	5	20	0.833
Weighted KNN	0.792	0.826	0.800	0.813	0.809	19	4	5	20	0.833
Cubic KNN	0.762	0.696	0.800	0.750	0.727	16	7	5	20	0.831
Kernel Naive Bayes	0.708	0.739	0.720	0.729	0.723	17	6	7	18	0.828
Cosine KNN	0.800	0.696	0.840	0.771	0.744	16	7	4	21	0.811
Medium Gaussian SVM	0.783	0.783	0.800	0.792	0.783	18	5	5	20	0.809
Fine Gaussian SVM	0.773	0.739	0.800	0.771	0.756	17	6	5	20	0.800
Binary GLM Logistic Regression	0.760	0.826	0.760	0.792	0.792	19	4	6	19	0.793
Gaussian Naive Bayes	0.818	0.391	0.920	0.667	0.529	9	14	2	23	0.772
Quadratic Discriminant	0.688	0.478	0.800	0.646	0.564	11	12	5	20	0.717
Fine Tree	0.682	0.652	0.720	0.688	0.667	15	8	7	18	0.708
Medium Tree	0.682	0.652	0.720	0.688	0.667	15	8	7	18	0.708
Coarse Tree	0.696	0.696	0.720	0.708	0.696	16	7	7	18	0.706
Fine KNN	0.667	0.696	0.680	0.688	0.681	16	7	8	17	0.688
Cubic SVM	0.500	0.044	0.960	0.521	0.080	1	22	1	24	0.115
Quadratic SVM	NaN	0.000	1.000	0.521	NaN	0	23	0	25	0.000
Coarse KNN	NaN	0.000	1.000	0.521	NaN	0	23	0	25	0.000

AUC, area under receiver operating characteristic curve; FN: False Negative; FP: False Positive; GLM: Generalized linear models; KNN: k-nearest neighbors; SVM: Support Vector Machines; TN: True Negative TP: True Positive

#### Predicting risk of epilepsy: tumor location plus radiomics

As shown in Table 4, the Linear SVM model yielded the best prediction performance based on radiomics features and tumor location features, as evidenced by the following metrics: Precision (0.955), recall (0.913), specificity (0.960), accuracy (0.938), F-1 score (0.933), and AUC (0.950). These findings indicate that the linear SVM model, incorporating both location and radiomics features, achieved the best overall prediction performance.

**Table 4:** Performance of seizure prediction models using radiomics features plus tumor location features for pediatric patients with supratentorial LGG

Classifier	Precision	Recall	Specificity	Accuracy	F1-Score	TP	FN	FP	TN	AUC
**Linear SVM**	**0.955**	**0.913**	**0.960**	**0.938**	**0.933**	**21**	**2**	**1**	**24**	**0.950**
Linear Discriminant	0.955	0.913	0.960	0.938	0.933	21	2	1	24	0.929
Medium Gaussian SVM	0.909	0.870	0.920	0.896	0.889	20	3	2	23	0.908
Fine Gaussian SVM	0.870	0.870	0.880	0.875	0.870	20	3	3	22	0.920
Coarse Gaussian SVM	0.840	0.913	0.840	0.875	0.875	21	2	4	21	0.920
Weighted KNN	0.870	0.870	0.880	0.875	0.870	20	3	3	22	0.903
Medium KNN	0.895	0.739	0.920	0.833	0.810	17	6	2	23	0.906
Cosine KNN	0.895	0.739	0.920	0.833	0.810	17	6	2	23	0.910
Fine Tree	0.792	0.826	0.800	0.813	0.809	19	4	5	20	0.813
Medium Tree	0.792	0.826	0.800	0.813	0.809	19	4	5	20	0.813
Coarse Tree	0.792	0.826	0.800	0.813	0.809	19	4	5	20	0.765
Binary GLM Logistic Regression	0.792	0.826	0.800	0.813	0.809	19	4	5	20	0.847
Fine KNN	0.818	0.783	0.840	0.813	0.800	18	5	4	21	0.811
Cubic KNN	0.889	0.696	0.920	0.813	0.781	16	7	2	23	0.902
Kernel Naive Bayes	0.833	0.652	0.880	0.771	0.732	15	8	3	22	0.889
Quadratic Discriminant	0.824	0.609	0.880	0.750	0.700	14	9	3	22	0.819
Gaussian Naive Bayes	0.867	0.565	0.920	0.750	0.684	13	10	2	23	0.810
Cubic SVM	1.000	0.087	1.000	0.563	0.160	2	21	0	25	0.087
Quadratic SVM	1.000	0.044	1.000	0.542	0.083	1	22	0	25	0.043
Coarse KNN	NaN	0.000	1.000	0.521	NaN	0	23	0	25	0.000

AUC, area under receiver operating characteristic curve; FN: False Negative; FP: False Positive; GLM: Generalized linear models; KNN: k-nearest neighbors; SVM: Support Vector Machines; TN: True Negative TP: True Positive

#### Top 8 features selected using (mRMR) algorithm

To ensure standardized feature extraction and analysis, the 206 radiomics features used in this study were selected based on guidelines published by the Image Biomarker Standardization Initiative (IBSI) (Supplemental Table). The mRMR algorithm identified the top 8 most important features (highlighted in red), including features related to tumor location (*n =* 2), shape (*n =* 2), grayscale intensity (*n =* 1), and texture (*n =* 3). Feature selection for the prediction of epilepsy risk was performed using leave-one-out cross-validation (LOOCV). The selected radiomics signatures proved highly effective in differentiating between glioma patients with and without epilepsy.

**Fig. 3:**
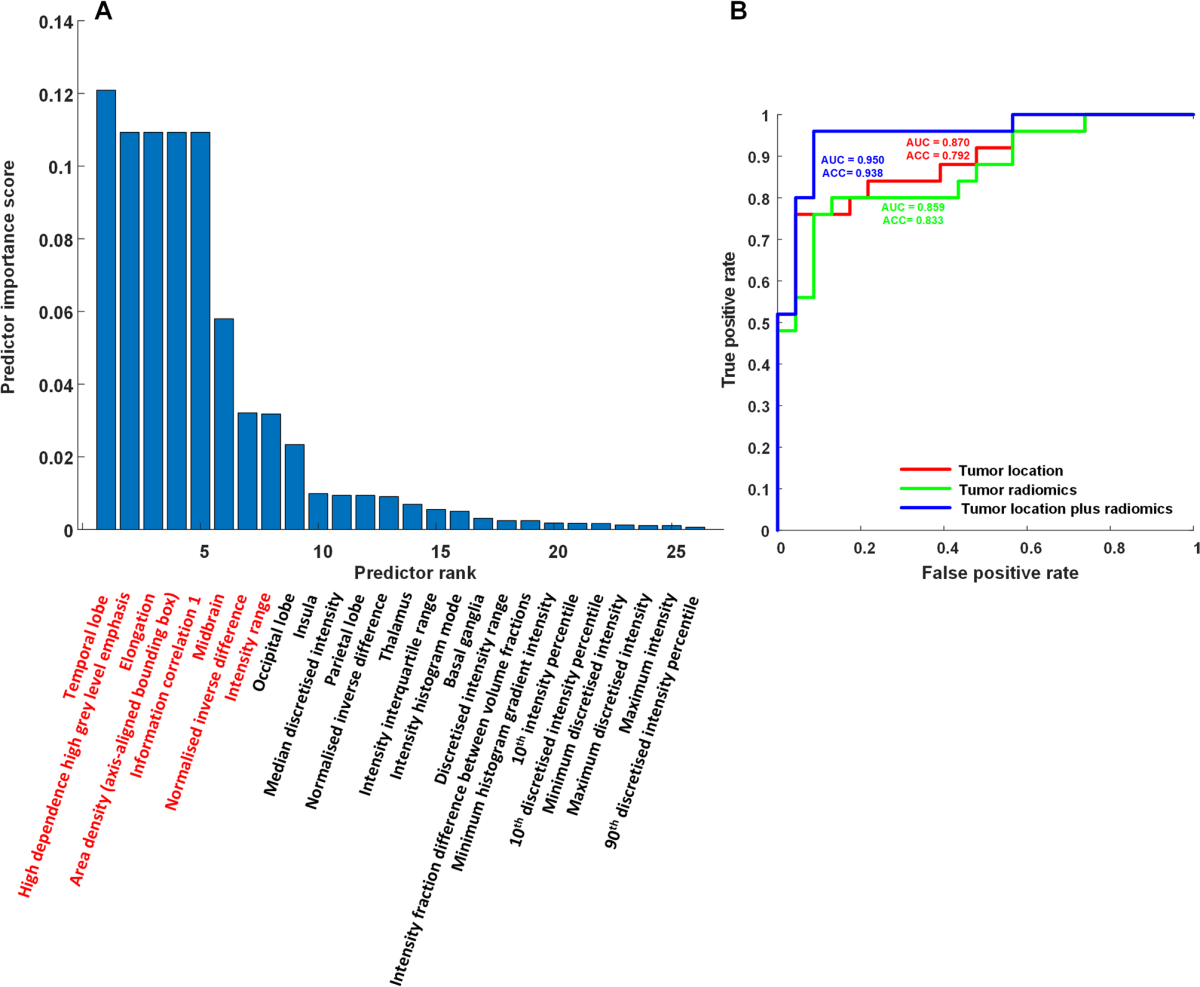
Importance scores of predictors selected from 216 features, including 10 tumor locations and 206 radiomics features: (**A**) The most important predictor was temporal lobe involvement (location), followed by high dependence high grey level emphasis (texture), elongation (shape), area density (axis-aligned bounding box, shape feature), information correlation 1 (Grey Level Co-occurrence Based Features), midbrain (location), normalized inverse difference (texture) and intensity range (intensity feature); (**B**) ROC curves depicting the validation performance of models based on tumor location alone, radiomic features alone, and the combination of both tumor location and radiomic features. Receiver operating characteristic curve (ROC), area under curve (AUC), and accuracy (ACC)

Figure 3A presents the top 8 most important characteristics included temporal lobe (location), high dependence high grey level emphasis (texture), elongation (shape), area density (axis-aligned bounding box, shape), information correlation 1 (Grey Level Co-occurrence Based Features), midbrain (location), normalized inverse difference (texture), and intensity range (intensity feature). Figure 3B presents the ROC curves from multivariate analysis along with the AUC and accuracy values for three models: (1) tumor location only, (2) radiomics only, and (3) a combination of tumor location and radiomics. The ROC curve revealed that tumor location plus radiomics model (represented by the blue line) achieved the highest accuracy and AUC.

## Discussion

Radiomics is a recent development that enables the transformation of medical images into quantitative biomarkers for prediction and decision support models [8]. Most of the MRI radiomics research on supratentorial glioma has focused on tumor grading, patient prognosis, molecular mutation subtyping, and predicting epilepsy in adult populations [9, 12, 21]. Although the incidence of high-grade glioma is higher among adult patients, the incidence of clinical epilepsy is higher among younger patients. To the best our knowledge, this is the first study focusing on epileptic seizure prediction among children with supratentorial LGG. Our Linear SVM model based on radiomics features plus tumor location data demonstrated outstanding performance in predicting the risk of epilepsy, achieving high accuracy of 0.938 with balanced accuracy rate of 85%, precision of 0.955, specificity of 0.960 and AUC 0.95. Our analysis identified eight key radiomics features from a total of 206, which could be used to identify pediatric LGG patients at risk of epilepsy, including features related to tumor location (*n* = 2), shape (*n* = 2), grayscale intensity (*n* = 1), and texture (*n* = 3).

Epileptic seizures often develop in children with glioma. Accumulating evidence in molecular science suggests that tumor growth stimulates seizures which in turn encourage tumor growth suggesting the two conditions may share common pathogenic mechanisms [22]. Glioma patients with chronic epileptic seizures present elevated glutamate signaling, altered peri-lesional immune reactivity, and increased of γ-aminobutyric acid (GABA) receptor concentrations [23, 24]. One hypothesis suggests that glioma cell mitosis and migration are promoted by intracellular chloride channel disruption and chloride accumulation in neurons leading to aberrant depolarization through GABA receptor activation, facilitating epileptic activity [25]. The molecular target of the rapamycin (mTOR) pathway and epigenetic abnormalities have also been implicated in the development of tumors and seizure onset [26]. This finding suggests that antitumor therapy may contribute to seizure control, while antiseizure medication could exert tumor-suppressive effects [27, 28]. Precisely targeted anti-seizure and anti-tumor therapies are essential for optimizing treatment outcomes in patients with both epilepsy and gliomas.

Our results demonstrated the effectiveness of the proposed radiomics model for the stratification of LGG patients according to seizure occurrence. Following multiple trials with various MRI sequences, including contrast-enhanced T1-weighted and T2-weighted sequences, it was determined that T2-FLAIR images were the best for distinguishing between seizure and non-seizure groups. T2-FLAIR has been reported as a biomarker of the poor survival of LGG [29], and T2-FLAIR mismatch sign has been reported as a specific biomarker in diagnosing of isocitrate dehydrogenase (IDH)–mutant 1p/19q non-codeleted LGGs in adults [30, 31], and B-Raf proto-oncogene, serine/threonine kinase (BRAF), and MYB or MYBL1 alternations related pediatric LGG [32, 33]. T2-FLAIR MRI have also been shown to provide precise glioma tumor information with spatial resolution exceeding that of T2WI, apparent diffusion coefficient (ADC) maps, and contrast-enhanced T1-weighted sequences [34, 35].

Among the 20 models assessed in this study, Linear SVM proved to be the most effective for epilepsy prediction, demonstrating the highest accuracy and precision. SVMs are a type of machine learning algorithm commonly used for both classification and regression tasks. SVMs have become widely adopted across diverse fields, including text categorization, image classification, and bioinformatics [36]. They are especially valued for their strong generalization capabilities, resilience to noise, and effectiveness in handling high-dimensional datasets. While several models including the Coarse Gaussian SVM, linear SVM was ultimately favored based on the following considerations. First, linear SVM has a simpler structure and fewer hyperparameters compared to kernel-based SVMs. This simplicity reduces the risk of overfitting, which is particularly important in small sample sizes like ours (*n* = 48). Second, linear SVM converged faster and required significantly less computational time than kernel-based models. Finally, linear SVM allows for easier interpretation and identification of the most influential features, which is critical for clinical translation, and demonstrates a balanced and robust performance in our study.

The mRMR algorithm was used primarily for screening and ranking with the aim of selecting the features that are most discriminative for target variables (e.g., classification labels) while minimizing redundancy among features [37, 38]. Unlike conventional statistical methods, mRMR feature selection can be tailored to weight features according to specific criteria, such as relevance or importance. This algorithm is more widely used than methods in the frequency-domain or 3D wavelet-transformed imaging techniques, particularly for feature selection in high-dimensionality data sets, such as gene expression data or medical imaging data.

Leave-on-out cross-validation (LOOCV) has emerged as a valuable tool for improving prediction performance in structured models [39]. This machine learning technique is particularly beneficial when working with small or unbalanced datasets, as it helps to reduce bias in performance estimation [40]. Cross-validation helps to reduce variance by providing a more accurate estimate of the model’s performance when applied to new data, whereas nested cross-validation offers a nearly unbiased estimate of the true error, further enhancing model generalizability [41].

Gray Matter Density (GMD) analysis enables the high-sensitivity detection. of correlations between the loss of gray matter and cerebrospinal fluid volume or cortical thickness [18, 19]. GMD makes it possible to assess regional neuronal injury, plasticity, and neuropathological heterogeneity [42]. GMD analysis has been widely applied in the study of epilepsy, stroke, and other neurodegenerative diseases [18, 42]. Our study revealed significant differences in GMD between pediatric LGG cases with epilepsy and those without. These findings enable the precise identification and measurement of GMD alterations, offering valuable insights into the differences between epilepsy and non- epilepsy groups in cases of pediatric supratentorial LGG.

The temporal lobe is far known most vulnerable lobe for occurrence of seizures [2, 43]. In this study, incorporating tumor location improved prediction precision from 0.800 to 0.955 and AUC from 0.855 to 0.95, differing from most adult studies, which did not include tumor location and focused on non-pediatric populations [12]. A high percentage of children with tumors in the temporal cortex experience epilepsy (60–83%), a rate far exceeding that associated with tumors in other cortical areas [44, 45]. Tumors situated in deeper structures, such as the basal ganglia and midbrain, are generally not associated with epilepsy [43]. In the current study, tumors involving multiple lobes (the basal ganglia and midbrain) were less likely to be associated with epilepsy.

This study identified 8 MRI radiomics features that are predictive of epilepsy, potentially reflecting alteration of tumor heterogeneity or homogeneity, or the tumor microenvironment [8, 12]. The radiomics predictors in this study include high dependence high grey level emphasis and area density, are biomarkers of tumor grey levels, heterogeneity and density. Previous radiomics studies have hypothesized that “uniformity” or tumor heterogeneity could serve as a predictor of epilepsy for dysembryoplastic neuroepithelial tumor (DNET) or tumor malignancy [46, 47]. Peritumoral edema has been linked to tumor recurrence or tumor progression in gliomas [48]. Unlike previous research, we also identified elongation shape as a key predictor. Peritumoral region is likely to contain the epileptogenic zone, and molecular pathway within the tumor may contribute to the primary etiologies of epileptogenesis. Previous studies have found an increased concentration of glutamate in both the tumor and peritumoral areas, which is significantly correlated with the presence of seizures, and GABA-A receptors in glioma cells induce depolarization instead of hyperpolarization [49]. However, the molecular pathways linked to radiomic features remain unclear. Further exploration into the mechanisms associated with epileptogenesis in LGG and their correlation with radiomics changes is recommended.

Radiomics has been shown to help bridge imaging features to clinical treatment. Our model could help be integrated into clinical workflows including preoperative analyzing routine T2-FLAIR MRI prior to surgery for seizure prediction, inform more aggressive resection strategies (e.g., extended margin) when feasible, or even guide intraoperative EEG mapping, further predict postoperative seizure likelihood and the need for ASM prophylaxis, and determine which patients require closer follow-ups, and tailor ASM regimens.

### Limitations

This study was a retrospective cohort study with a small sample size. We did not include infratentorial and high-grade gliomas due to their different nature, genetic background, and the low incidence of preoperative seizures. However, this selection bias might limit the generalizability of our findings. One confounding factor is that most patients with seizures received ASM treatment preoperatively, which may have altered the tumor environment and affected the tumor’s properties. We did not analyze tumor molecular subtypes due to the small cohort of patients. Future research should focus on understanding radiomics in relation to molecular subtypes across a larger cohort within various tumor histology. While leave-one-out cross-validation (LOOCV) mitigates this to some extent, the results should be interpreted cautiously. A larger, multi-center validation cohort is essential to confirm generalizability.

## Conclusions

This study identified 8 radiomics features that are predictive of epilepsy risk in children with LGG. The features identified by the mRMR algorithm included features related to 2 tumor locations, 2 shapes, 1 grayscale intensity, and 3 texture. When combined with significant tumor locations, the proposed prediction model makes it possible to determine with a high degree of precision whether epilepsies are associated with supratentorial LGG. Understanding the impact of epilepsy on pediatric brain tumors is crucial to the accuracy of diagnosis and pretherapeutic decisions. The radiomics and location biomarkers identified in this study can help facilitate the early identification of children at the highest risk, enabling enables targeted treatment while minimizing exposure to potentially toxic or unnecessary therapies in patients at minimal risk.

## Electronic supplementary material

Below is the link to the electronic supplementary material.

Supplementary Material 1

**Supplementary Material 2**: **Supplementary fig. 1**: Confusion matrix illustrating the classification performance of the best-performing model. The matrix includes true positives (TP = 21), false negatives (FN = 2), false positives (FP = 1), and true negatives (TN = 24). Positive predictive value (PPV) and negative predictive value (NPV) were calculated as 0.955 and 0.923, respectively, providing additional context for the model’s diagnostic performance.

## Data Availability

The datasets used and analyzed during the current study are available from the corresponding author on reasonable request.

## Abbreviations

3-D: Three dimensional
AUC: Area under curve
BRAF: B-raf proto-oncogene, serine/threonine kinase
EEG: Electroencephalogram
FLAIR: Fluid-attenuated inversion recovery
FWHM: Full width at half maximum
GABA: γ-aminobutyric acid
GMD: Gray matter density
IBSI: Image biomarker standardization initiative
IDH: Isocitrate dehydrogenase
LGG: Low grade gliomas
LOOCV: Leave-one-out cross-validation
MRI: Magnetic resonance imaging
mRMR: Minimum redundancy maximum relevance
mTOR: Molecular target of the rapamycin
ROC: Receiver operating characteristic curve
ROIs: Region of interests
SVM: Support vector machine
T2W: T2-weighted
WHO: World health organization
