# Supplementary material for: Morphometric and radiomics analysis toward the prediction of epilepsy associated with supratentorial low-grade glioma in children
Source: Cancer Imaging. 2025 May 19;25:63. doi: 10.1186/s40644-025-00881-1 (PMC12090388; doi:10.1186/s40644-025-00881-1)
Supplement: Supplementary file 1 — Supplementary Material 1 [file 40644_2025_881_MOESM1_ESM.docx]

**Supplementary Table 1** IBSI (Image Biomarker Standardization Initiative) Standard and Radiomics Function Feature Correspondences. The red annotations indicate the top 8 features, which were selected using the minimum redundancy maximum relevance (mRMR) algorithm.

|  |  |  | **With epilepsy (n=23)** | | **Without epilepsy (n=25)** | |  |  |
| --- | --- | --- | --- | --- | --- | --- | --- | --- |
|  | **IBSI Feature** | **Corresponding Feature** | **MEAN** | **STD** | **MEAN** | **STD** |  | ***p*** |
|  | **Local Intensity Features** | | | | | |  |  |
|  | Local intensity peak | LocalIntensityPeak3D | 0.970 | 0.871 | 0.830 | 0.712 |  | 0.312 |
|  | Global intensity peak | GlobalIntensityPeak3D | 1.185 | 0.612 | 0.960 | 0.625 |  | 0.122 |
|  | **Intensity Based Statistical Features** | | | | | |  |  |
|  | Mean intensity | MeanIntensity3D | -0.096 | 0.093 | -0.109 | 0.082 |  | 0.409 |
|  | Intensity variance | IntensityVariance3D | 0.933 | 0.207 | 0.916 | 0.146 |  | 0.606 |
|  | Intensity skewness | IntensitySkewness3D | -0.344 | 0.458 | -0.299 | 0.453 |  | 0.741 |
|  | (Excess) intensity kurtosis | IntensityKurtosis3D | 0.158 | 0.598 | 0.014 | 0.747 |  | 0.353 |
|  | Median intensity | MedianIntensity3D | 0.000 | 0.000 | 0.000 | 0.000 |  | NaN |
|  | Minimum intensity | MinimumIntensity3D | -2.652 | 0.573 | -2.600 | 0.645 |  | 0.869 |
|  | 10^th^ intensity percentile | TenthIntensityPercentile3D | -1.217 | 0.422 | -1.320 | 0.476 |  | 0.437 |
|  | 90^th^ intensity percentile | NinetiethIntensityPercentile3D | 1.000 | 0.000 | 1.000 | 0.000 |  | NaN |
|  | Maximum intensity | MaximumIntensity3D | 2.087 | 0.417 | 2.000 | 0.408 |  | 0.474 |
|  | Intensity interquartile range | IntensityInterquartileRange3D | 1.391 | 0.722 | 1.520 | 0.586 |  | 0.617 |
|  | **Intensity range** | **IntensityRange3D** | **4.739** | **0.689** | **4.600** | **0.866** |  | **0.528** |
|  | Intensity-based mean absolute deviation | MeanAbsoluteDeviation3D | 0.724 | 0.117 | 0.733 | 0.081 |  | 1.000 |
|  | Intensity-based robust mean absolute deviation | RobustMeanAbsoluteDeviation3D | 0.590 | 0.098 | 0.616 | 0.113 |  | 0.470 |
|  | Intensity-based median absolute deviation | MedianAbsoluteDeviation3D | 0.689 | 0.107 | 0.694 | 0.081 |  | 0.577 |
|  | Intensity-based coefficient of variation | CoefficientOfVariation3D | -6.756 | 22.037 | 4.977 | 71.613 |  | 0.433 |
|  | Intensity-based quartile coefficient of dispersion | QuartileCoefficientOfDispersion3D | 652173.630 | 486985.090 | 599999.750 | 500000.250 |  | 0.781 |
|  | Intensity-based energy | IntensityEnergy3D | 32979.219 | 34611.945 | 19636.199 | 23770.447 |  | 0.149 |
|  | Root mean square intensity | RootMeanSquare3D | 0.968 | 0.117 | 0.963 | 0.078 |  | 0.665 |
|  | **Intensity Histogram Features** | | | | | |  |  |
|  | Mean discretized intensity | MeanDiscretizedIntensity3D | 1.703 | 0.276 | 1.629 | 0.322 |  | 0.187 |
|  | Discretized intensity variance | DiscretizedIntensityVariance3D | 0.223 | 0.057 | 0.223 | 0.038 |  | 0.695 |
|  | Discretized intensity skewness | DiscretizedIntensitySkewness3D | -0.797 | 0.522 | -0.683 | 0.461 |  | 0.550 |
|  | (Excess) discretized intensity kurtosis | DiscretizedIntensityKurtosis3D | -0.900 | 0.974 | -1.188 | 0.557 |  | 0.332 |
|  | Median discretized intensity | MedianDiscretizedIntensity3D | 1.957 | 0.209 | 1.880 | 0.332 |  | 0.354 |
|  | Minimum discretized intensity | MinimumDiscretizedIntensity3D | 0.957 | 0.209 | 0.920 | 0.277 |  | 0.623 |
|  | 10th discretized intensity percentile | TenthDiscretizedIntensityPercentile3D | 1.043 | 0.367 | 0.960 | 0.351 |  | 0.429 |
|  | 90th discretized intensity percentile | NinetiethDiscretizedIntensityPercentile3D | 2.043 | 0.367 | 2.000 | 0.408 |  | 0.711 |
|  | Maximum discretized intensity | MaximumDiscretizedIntensity3D | 2.087 | 0.417 | 2.000 | 0.408 |  | 0.474 |
|  | Intensity histogram mode | IntensityHistogramMode3D | 1.957 | 0.209 | 1.880 | 0.332 |  | 0.354 |
|  | Discretized intensity interquartile range | DiscretizedIntensityInterquartileRange3D | 0.826 | 0.388 | 0.840 | 0.374 |  | 0.911 |
|  | Discretized intensity range | DiscretizedIntensityRange3D | 1.130 | 0.344 | 1.080 | 0.277 |  | 0.585 |
|  | Intensity histogram mean absolute deviation | IntensityHistogramMeanAbsoluteDeviation3D | 0.419 | 0.064 | 0.428 | 0.040 |  | 0.710 |
|  | Intensity histogram robust mean absolute deviation | IntensityHistogramRobustMeanAbsoluteDeviation3D | 0.415 | 0.059 | 0.428 | 0.041 |  | 0.509 |
|  | Intensity histogram median absolute deviation | IntensityHistogramMedianAbsoluteDeviation3D | 0.310 | 0.072 | 0.320 | 0.053 |  | 0.665 |
|  | Intensity histogram coefficient of variation | IntensityHistogramCoeffcientOfVariation3D | 0.289 | 0.101 | 0.306 | 0.105 |  | 0.283 |
|  | Intensity histogram quartile coefficient of dispersion | IntensityHistogramQuartileCoeffcientOfDispersion3D | 0.293 | 0.201 | 0.296 | 0.193 |  | 0.908 |
|  | Discretized intensity entropy | DiscretizedIntensityEntropy3D | 0.914 | 0.164 | 0.918 | 0.112 |  | 0.695 |
|  | Discretized intensity uniformity | DiscretizedIntensityUniformity3D | 0.575 | 0.070 | 0.567 | 0.046 |  | 0.695 |
|  | Maximum histogram gradient | MaximumHistogramGradient3D | 14690.695 | 16244.660 | 7415.120 | 8156.006 |  | 0.137 |
|  | Maximum histogram gradient intensity | MaximumHistogramGradientIntensity3D | 1.000 | 0.000 | 1.000 | 0.000 |  | NaN |
|  | Minimum histogram gradient | MinimumHistogramGradient3D | 10979.565 | 18120.420 | 5840.600 | 8853.313 |  | 0.375 |
|  | Minimum histogram gradient intensity | MinimumHistogramGradientIntensity3D | 1.261 | 0.689 | 1.160 | 0.554 |  | 0.585 |
|  | **Intensity Volume Histogram Features** | | | | | |  |  |
|  | Volume at intensity fraction | TenPercentVolumeFraction3D | 0.970 | 0.023 | 0.956 | 0.067 |  | 0.757 |
|  | Volume at intensity fraction | NinetyPercentVolumeFraction3D | 0.033 | 0.070 | 0.044 | 0.078 |  | 0.509 |
|  | Intensity at volume fraction | TenPercentIntensityFraction3D | 730.826 | 168.374 | 707.680 | 216.728 |  | 0.859 |
|  | Intensity at volume fraction | NinetyPercentIntensityFraction3D | 299.783 | 78.524 | 269.760 | 123.887 |  | 0.346 |
|  | Volume fraction difference between intensity fractions | VolumeFractionDifference3D | 0.937 | 0.072 | 0.912 | 0.115 |  | 0.483 |
|  | Intensity fraction difference between volume fractions | IntensityFractionDifference3D | 431.043 | 168.134 | 437.920 | 237.435 |  | 0.306 |
|  | **Shape Features** | | | | | |  |  |
|  | Volume (mesh) | VolumeMesh3D | 36441.000 | 37231.270 | 22365.480 | 27497.199 |  | 0.161 |
|  | Volume (voxel counting) | VolumeVoxelCount3D | 36497.391 | 37257.141 | 22412.561 | 27514.578 |  | 0.161 |
|  | Surface area (mesh) | SurfaceAreaMesh3D | 8344.398 | 7118.585 | 5387.888 | 4343.625 |  | 0.180 |
|  | Surface to volume ratio | SurfaceVolumeRatio3D | 0.305 | 0.111 | 0.352 | 0.132 |  | 0.231 |
|  | Compactness 1 | Compactness1_3D | 0.026 | 0.005 | 0.027 | 0.004 |  | 0.522 |
|  | Compactness 2 | Compactness2_3D | 0.253 | 0.095 | 0.270 | 0.087 |  | 0.522 |
|  | Spherical disproportion | SphericalDisproportion3D | 1.644 | 0.267 | 1.583 | 0.176 |  | 0.522 |
|  | Sphericity | Sphericity3D | 0.621 | 0.087 | 0.639 | 0.070 |  | 0.522 |
|  | Asphericity | Asphericity3D | 0.644 | 0.267 | 0.583 | 0.176 |  | 0.522 |
|  | Centre of mass shift | CentreOfMassShift3D | 0.969 | 0.630 | 0.685 | 0.732 |  | 0.029 |
|  | Maximum 3D diameter | Maximum3dDiameter3D | 58.015 | 27.363 | 51.615 | 19.585 |  | 0.650 |
|  | Major axis length | MajorAxisLength3D | 45.760 | 19.786 | 44.663 | 17.410 |  | 0.901 |
|  | Minor axis length | MinorAxisLength3D | 36.323 | 15.178 | 28.255 | 10.147 |  | 0.069 |
|  | Least axis length | LeastAxisLength3D | 26.592 | 9.360 | 22.650 | 8.961 |  | 0.208 |
|  | **Elongation** | **Elongation3D** | **0.800** | **0.096** | **0.664** | **0.178** |  | **0.004** |
|  | Flatness | Flatness3D | 0.600 | 0.088 | 0.535 | 0.170 |  | 0.117 |
|  | Volume density (axis-aligned bounding box) | VolumeDensityAABB_3D | 0.375 | 0.083 | 0.326 | 0.103 |  | 0.127 |
|  | **Area density (axis-aligned bounding box)** | **AreaDensityAABB_3D** | **0.661** | **0.067** | **0.575** | **0.097** |  | **<0.001** |
|  | Volume density (approximate enclosing ellipsoid) | VolumeDensityAEE_3D | 1.114 | 0.137 | 1.055 | 0.230 |  | 0.635 |
|  | Area density (approximate enclosing ellipsoid) | AreaDensityAEE_3D | 1.646 | 0.194 | 1.594 | 0.248 |  | 0.741 |
|  | Volume density (convex hull) | VolumeDensityConvexHull3D | 0.684 | 0.088 | 0.656 | 0.131 |  | 0.757 |
|  | Area density (convex hull) | AreaDensityConvexHull3D | 1.136 | 0.081 | 1.051 | 0.120 |  | 0.018 |
|  | Integrated intensity | IntegratedIntensity3D | 39311596.000 | 98234696.000 | 10909544.000 | 9863715.000 |  | 0.122 |
|  | **Grey Level Co-occurrence Based Features** | | | | | |  |  |
|  | Joint maximum | JointMaximumAveraged3D | 0.014 | 0.017 | 0.016 | 0.017 |  | 0.496 |
|  | Joint average | JointAverageAveraged3D | 64.638 | 132.117 | 31.008 | 14.659 |  | 0.132 |
|  | Joint variance | JointVarianceAveraged3D | 1892.174 | 8158.617 | 135.110 | 177.312 |  | 0.154 |
|  | Joint entropy | JointEntropyAveraged3D | 9.501 | 2.009 | 8.993 | 1.337 |  | 0.386 |
|  | Difference average | DifferenceAverageAveraged3D | 6.987 | 12.079 | 4.226 | 2.239 |  | 0.901 |
|  | Difference variance | DifferenceVarianceAveraged3D | 161.386 | 634.817 | 21.350 | 26.151 |  | 0.445 |
|  | Difference entropy | DifferenceEntropyAveraged3D | 3.601 | 1.161 | 3.423 | 0.648 |  | 0.820 |
|  | Sum average | SumAverageAveraged3D | 129.277 | 264.233 | 62.015 | 29.318 |  | 0.132 |
|  | Sum variance | SumVarianceAveraged3D | 7212.784 | 31214.730 | 495.632 | 672.977 |  | 0.143 |
|  | Sum entropy | SumEntropyAveraged3D | 6.372 | 1.227 | 5.886 | 0.784 |  | 0.137 |
|  | Angular second moment | AngularSecondMomentAveraged3D | 0.005 | 0.008 | 0.005 | 0.005 |  | 0.536 |
|  | Contrast | ContrastAveraged3D | 355.914 | 1420.536 | 44.809 | 52.854 |  | 0.665 |
|  | Dissimilarity | DissimilarityAveraged3D | 6.987 | 12.079 | 4.226 | 2.239 |  | 0.901 |
|  | Inverse difference | InverseDifferenceAveraged3D | 0.358 | 0.137 | 0.357 | 0.090 |  | 0.757 |
|  | Normalized inverse difference | NormalizedInverseDifferenceAveraged3D | 0.944 | 0.013 | 0.933 | 0.014 |  | 0.005 |
|  | Inverse difference moment | InverseDifferenceMomentAveraged3D | 0.285 | 0.145 | 0.277 | 0.099 |  | 0.680 |
|  | Normalized inverse difference moment | NormalizedInverseDifferenceMomentAveraged3D | 0.992 | 0.004 | 0.989 | 0.004 |  | 0.035 |
|  | Inverse variance | InverseVarianceAveraged3D | 0.268 | 0.109 | 0.269 | 0.079 |  | 0.773 |
|  | Correlation | CorrelationAveraged3D | 0.847 | 0.078 | 0.782 | 0.093 |  | 0.013 |
|  | Autocorrelation | AutoCorrelationAveraged3D | 22588.582 | 99186.227 | 1280.550 | 1232.834 |  | 0.149 |
|  | Cluster tendency | ClusterTendencyAveraged3D | 7212.784 | 31214.730 | 495.632 | 672.977 |  | 0.143 |
|  | Cluster shade | ClusterShadeAveraged3D | 164260.690 | 841574.630 | -3203.841 | 19290.775 |  | 0.458 |
|  | Cluster prominence | ClusterProminenceAveraged3D | 2749543900.000 | 13172920000.000 | 1578512.000 | 3633205.500 |  | 0.132 |
|  | **Information correlation 1** | **InformationCorrelation1Averaged3D** | **-0.251** | **0.065** | **-0.192** | **0.059** |  | **0.001** |
|  | Information correlation 2 | InformationCorrelation2Averaged3D | 0.948 | 0.035 | 0.890 | 0.069 |  | 0.001 |
|  | Joint maximum | JointMaximumMerged3D | 0.014 | 0.017 | 0.015 | 0.017 |  | 0.606 |
|  | Joint average | JointAverageMerged3D | 64.634 | 132.112 | 31.003 | 14.656 |  | 0.132 |
|  | Joint variance | JointVarianceMerged3D | 1893.051 | 8162.374 | 135.260 | 177.492 |  | 0.154 |
|  | Joint entropy | JointEntropyMerged3D | 9.758 | 2.350 | 9.138 | 1.392 |  | 0.433 |
|  | Difference average | DifferenceAverageMerged3D | 6.953 | 12.034 | 4.203 | 2.226 |  | 0.869 |
|  | Difference variance | DifferenceVarianceMerged3D | 166.548 | 654.786 | 21.998 | 27.001 |  | 0.470 |
|  | Difference entropy | DifferenceEntropyMerged3D | 3.638 | 1.165 | 3.461 | 0.649 |  | 0.820 |
|  | Sum average | SumAverageMerged3D | 129.268 | 264.223 | 62.006 | 29.313 |  | 0.132 |
|  | Sum variance | SumVarianceMerged3D | 7218.800 | 31238.793 | 496.618 | 673.954 |  | 0.143 |
|  | Sum entropy | SumEntropyMerged3D | 6.389 | 1.237 | 5.901 | 0.787 |  | 0.143 |
|  | Angular second moment | AngularSecondMomentMerged3D | 0.005 | 0.008 | 0.005 | 0.005 |  | 0.536 |
|  | Contrast | ContrastMerged3D | 353.402 | 1411.477 | 44.420 | 52.431 |  | 0.665 |
|  | Dissimilarity | DissimilarityMerged3D | 6.953 | 12.034 | 4.203 | 2.226 |  | 0.869 |
|  | Inverse difference | InverseDifferenceMerged3D | 0.359 | 0.137 | 0.359 | 0.090 |  | 0.773 |
|  | **Normalized inverse difference** | **NormalizedInverseDifferenceMerged3D** | **0.944** | **0.013** | **0.933** | **0.014** |  | **0.005** |
|  | Inverse difference moment | InverseDifferenceMomentMerged3D | 0.286 | 0.145 | 0.278 | 0.099 |  | 0.680 |
|  | Normalized inverse difference moment | NormalizedInverseDifferenceMomentMerged3D | 0.992 | 0.004 | 0.989 | 0.004 |  | 0.035 |
|  | Inverse variance | InverseVarianceMerged3D | 0.269 | 0.109 | 0.270 | 0.079 |  | 0.773 |
|  | Correlation | CorrelationMerged3D | 0.849 | 0.076 | 0.785 | 0.092 |  | 0.013 |
|  | Autocorrelation | AutoCorrelationMerged3D | 22588.547 | 99186.617 | 1280.448 | 1232.584 |  | 0.149 |
|  | Cluster tendency | ClusterTendencyMerged3D | 7218.800 | 31238.793 | 496.618 | 673.954 |  | 0.143 |
|  | Cluster shade | ClusterShadeMerged3D | 163104.450 | 836349.000 | -3211.366 | 19391.850 |  | 0.458 |
|  | Cluster prominence | ClusterProminenceMerged3D | 2755041800.000 | 13199237000.000 | 1584258.900 | 3641000.300 |  | 0.132 |
|  | Information correlation 1 | InformationCorrelation1Merged3D | -0.212 | 0.070 | -0.165 | 0.059 |  | 0.006 |
|  | Information correlation 2 | InformationCorrelation2Merged3D | 0.934 | 0.040 | 0.875 | 0.069 |  | 0.002 |
|  | **Grey Level Run Length Based Features** | | | | | |  |  |
|  | Short runs emphasis | ShortRunsEmphasisAveraged3D | 0.913 | 0.069 | 0.923 | 0.042 |  | 0.592 |
|  | Long runs emphasis | LongRunsEmphasisAveraged3D | 1.745 | 1.417 | 1.525 | 0.577 |  | 0.650 |
|  | Low grey level run emphasis | LowGrayLevelRunEmphasisAveraged3D | 0.006 | 0.006 | 0.007 | 0.007 |  | 0.726 |
|  | High grey level run emphasis | HighGrayLevelRunEmphasisAveraged3D | 22566.285 | 99183.688 | 1270.436 | 1193.945 |  | 0.149 |
|  | Short run low grey level emphasis | ShortRunLowGrayLevelEmphasisAveraged3D | 0.005 | 0.005 | 0.006 | 0.006 |  | 0.726 |
|  | Short run high grey level emphasis | ShortRunHighGrayLevelEmphasisAveraged3D | 22386.484 | 98833.734 | 1192.534 | 1136.443 |  | 0.161 |
|  | Long run low grey level emphasis | LongRunLowGrayLevelEmphasisAveraged3D | 0.013 | 0.031 | 0.013 | 0.029 |  | 0.804 |
|  | Long run high grey level emphasis | LongRunHighGrayLevelEmphasisAveraged3D | 23496.238 | 100695.950 | 1727.205 | 1541.567 |  | 0.069 |
|  | Grey level non-uniformity | GrayLevelNonUniformityAveraged3D | 1207.649 | 1763.985 | 798.962 | 1169.412 |  | 0.665 |
|  | Normalized grey level non-uniformity | NormalizedGrayLevelNonUniformityAveraged3D | 0.034 | 0.022 | 0.040 | 0.021 |  | 0.248 |
|  | Run length non-uniformity | RunLengthNonUniformityAveraged3D | 22515.266 | 19937.311 | 14682.369 | 14148.569 |  | 0.117 |
|  | Normalized run length non-uniformity | NormalizedRunLengthNonUniformityAveraged3D | 0.810 | 0.120 | 0.824 | 0.080 |  | 0.592 |
|  | Run percentage | RunPercentageAveraged3D | 0.881 | 0.090 | 0.893 | 0.059 |  | 0.550 |
|  | Grey level variance | GrayLevelVarianceAveraged3D | 2014.096 | 8709.539 | 143.822 | 179.821 |  | 0.194 |
|  | Run length variance | RunLengthVarianceAveraged3D | 0.336 | 0.729 | 0.231 | 0.318 |  | 0.606 |
|  | Run entropy | RunEntropyAveraged3D | 6.056 | 0.954 | 5.578 | 0.608 |  | 0.037 |
|  | Short runs emphasis | ShortRunsEmphasisMerged3D | 0.915 | 0.066 | 0.924 | 0.040 |  | 0.592 |
|  | Long runs emphasis | LongRunsEmphasisMerged3D | 1.635 | 0.974 | 1.487 | 0.472 |  | 0.577 |
|  | Low grey level run emphasis | LowGrayLevelRunEmphasisMerged3D | 0.006 | 0.006 | 0.007 | 0.007 |  | 0.726 |
|  | High grey level run emphasis | HighGrayLevelRunEmphasisMerged3D | 22566.436 | 99183.648 | 1270.605 | 1194.065 |  | 0.149 |
|  | Short run low grey level emphasis | ShortRunLowGrayLevelEmphasisMerged3D | 0.005 | 0.005 | 0.006 | 0.006 |  | 0.726 |
|  | Short run high grey level emphasis | ShortRunHighGrayLevelEmphasisMerged3D | 22387.516 | 98833.664 | 1193.480 | 1136.620 |  | 0.161 |
|  | Long run low grey level emphasis | LongRunLowGrayLevelEmphasisMerged3D | 0.011 | 0.024 | 0.013 | 0.029 |  | 0.788 |
|  | Long run high grey level emphasis | LongRunHighGrayLevelEmphasisMerged3D | 23475.766 | 100699.800 | 1712.546 | 1541.651 |  | 0.073 |
|  | Grey level non-uniformity | GrayLevelNonUniformityMerged3D | 15690.414 | 22913.764 | 10376.454 | 15182.744 |  | 0.665 |
|  | Normalized grey level non-uniformity | NormalizedGrayLevelNonUniformityMerged3D | 0.034 | 0.022 | 0.040 | 0.021 |  | 0.248 |
|  | Run length non-uniformity | RunLengthNonUniformityMerged3D | 292178.690 | 258828.730 | 190480.160 | 183197.950 |  | 0.117 |
|  | Normalized run length non-uniformity | NormalizedRunLengthNonUniformityMerged3D | 0.811 | 0.118 | 0.825 | 0.079 |  | 0.592 |
|  | Run percentage | RunPercentageMerged3D | 0.881 | 0.090 | 0.893 | 0.059 |  | 0.550 |
|  | Grey level variance | GrayLevelVarianceMerged3D | 2014.124 | 8709.533 | 143.845 | 179.898 |  | 0.194 |
|  | Run length variance | RunLengthVarianceMerged3D | 0.287 | 0.526 | 0.214 | 0.262 |  | 0.606 |
|  | Run entropy | RunEntropyMerged3D | 6.066 | 0.957 | 5.592 | 0.610 |  | 0.041 |
|  | **Grey Level Size Zone Based Features** | | | | | |  |  |
|  | Small zone emphasis | SmallZoneEmphasis3D | 0.640 | 0.122 | 0.629 | 0.082 |  | 1.000 |
|  | Large zone emphasis | LargeZoneEmphasis3D | 69597.742 | 319313.590 | 10555.963 | 40686.594 |  | 0.726 |
|  | Low grey level zone emphasis | LowGrayLevelZoneEmphasis3D | 0.013 | 0.022 | 0.011 | 0.013 |  | 0.563 |
|  | High grey level zone emphasis | HighGrayLevelZoneEmphasis3D | 22481.273 | 99608.703 | 1163.640 | 1081.130 |  | 0.173 |
|  | Small zone low grey level emphasis | SmallZoneLowGrayLevelEmphasis3D | 0.008 | 0.009 | 0.008 | 0.009 |  | 0.458 |
|  | Small zone high grey level emphasis | SmallZoneHighGrayLevelEmphasis3D | 20973.967 | 95025.320 | 773.727 | 779.040 |  | 0.208 |
|  | Large zone low grey level emphasis | LargeZoneLowGrayLevelEmphasis3D | 1019.063 | 4848.632 | 63.604 | 253.496 |  | 0.741 |
|  | Large zone high grey level emphasis | LargeZoneHighGrayLevelEmphasis3D | 6760225.500 | 24967894.000 | 2184282.300 | 7360917.000 |  | 0.154 |
|  | Grey level non-uniformity | GrayLevelNonUniformity3D | 151.662 | 139.886 | 133.360 | 115.713 |  | 0.741 |
|  | Normalized grey level non-uniformity | NormalizedGrayLevelNonUniformity3D | 0.026 | 0.015 | 0.032 | 0.013 |  | 0.107 |
|  | Zone size non-uniformity | ZoneSizeNonUniformity3D | 3492.392 | 5467.380 | 2169.304 | 2484.213 |  | 0.216 |
|  | Normalized zone size non-uniformity | NormalizedZoneSizeNonUniformity3D | 0.398 | 0.149 | 0.376 | 0.087 |  | 1.000 |
|  | Zone percentage | ZonePercentage3D | 0.320 | 0.227 | 0.303 | 0.148 |  | 0.820 |
|  | Grey level variance | GrayLevelVariance3D | 2051.310 | 8825.287 | 146.710 | 145.184 |  | 0.107 |
|  | Zone size variance | ZoneSizeVariance3D | 68950.203 | 316350.560 | 10490.065 | 40467.793 |  | 0.726 |
|  | Zone size entropy | ZoneSizeEntropy3D | 7.699 | 0.628 | 7.295 | 0.424 |  | 0.013 |
|  | **Grey Level Distance Zone Based Features** | | | | | |  |  |
|  | Small distance emphasis | SmallDistanceEmphasis3D | 0.543 | 0.141 | 0.604 | 0.144 |  | 0.167 |
|  | Large distance emphasis | LargeDistanceEmphasis3D | 14.355 | 13.133 | 11.026 | 9.776 |  | 0.239 |
|  | Low grey level zone emphasis | LowGrayLevelZoneDistanceEmphasis3D | 0.013 | 0.022 | 0.011 | 0.013 |  | 0.563 |
|  | High grey level zone emphasis | HighGrayLevelZoneDistanceEmphasis3D | 22481.273 | 99608.703 | 1163.640 | 1081.130 |  | 0.173 |
|  | Small distance low grey level emphasis | SmallDistanceLowGrayLevelEmphasis3D | 0.010 | 0.021 | 0.009 | 0.011 |  | 0.302 |
|  | Small distance high grey level emphasis | SmallDistanceHighGrayLevelEmphasis3D | 6216.619 | 25981.752 | 580.792 | 498.887 |  | 0.187 |
|  | Large distance low grey level emphasis | LargeDistanceLowGrayLevelEmphasis3D | 0.091 | 0.134 | 0.068 | 0.084 |  | 0.695 |
|  | Large distance high grey level emphasis | LargeDistanceHighGrayLevelEmphasis3D | 544768.630 | 2512591.500 | 15801.678 | 22894.938 |  | 0.112 |
|  | Grey level non-uniformity | GrayLevelDistanceNonUniformity3D | 151.662 | 139.886 | 133.360 | 115.713 |  | 0.741 |
|  | Normalized grey level non-uniformity | NormalizedGrayLevelDistanceNonUniformity3D | 0.026 | 0.015 | 0.032 | 0.013 |  | 0.107 |
|  | Zone distance non-uniformity | ZoneDistanceNonUniformity3D | 1775.786 | 1480.805 | 1290.887 | 777.905 |  | 0.421 |
|  | Normalized zone distance non-uniformity | NormalizedZoneDistanceNonUniformity3D | 0.308 | 0.136 | 0.367 | 0.147 |  | 0.194 |
|  | Zone percentage | ZoneDistancePercentage3D | 0.320 | 0.227 | 0.303 | 0.148 |  | 0.820 |
|  | Grey level variance | GrayLevelDistanceVariance3D | 2051.310 | 8825.287 | 146.710 | 145.184 |  | 0.107 |
|  | Zone distance variance | ZoneDistanceVariance3D | 5.896 | 5.686 | 4.602 | 4.794 |  | 0.208 |
|  | Zone distance entropy | ZoneDistanceEntropy3D | 7.847 | 1.457 | 7.156 | 1.106 |  | 0.083 |
|  | **Neighbourhood Grey Tone Difference Based Features** | | | | | |  |  |
|  | Coarseness | Coarseness3D | 0.002 | 0.001 | 0.002 | 0.002 |  | 0.522 |
|  | Contrast | Contrast3D | 0.249 | 0.343 | 0.177 | 0.143 |  | 0.967 |
|  | Busyness | Busyness3D | 2.959 | 9.368 | 1.765 | 3.161 |  | 0.650 |
|  | Complexity | Complexity3D | 708190.060 | 3353565.500 | 4900.726 | 6222.044 |  | 0.342 |
|  | Strength | Strength3D | 22.282 | 88.043 | 2.565 | 3.122 |  | 0.665 |
|  | **Neighbouring Grey Level Dependence Based Features** | | | | | |  |  |
|  | Low dependence emphasis | LowDependenceEmphasis3D | 0.294 | 0.204 | 0.274 | 0.123 |  | 0.710 |
|  | High dependence emphasis | HighDependenceEmphasis3D | 28.983 | 38.801 | 22.795 | 22.234 |  | 0.536 |
|  | Low grey level count emphasis | LowGrayLevelCountEmphasis3D | 0.005 | 0.005 | 0.007 | 0.010 |  | 0.710 |
|  | High grey level count emphasis | HighGrayLevelCountEmphasis3D | 22571.969 | 99148.555 | 1278.776 | 1210.100 |  | 0.143 |
|  | Low dependence low grey level emphasis | LowDependenceLowGrayLevelEmphasis3D | 0.002 | 0.001 | 0.002 | 0.001 |  | 0.073 |
|  | Low dependence high grey level emphasis | LowDependenceHighGrayLevelEmphasis3D | 19481.869 | 90074.266 | 420.824 | 503.432 |  | 0.445 |
|  | High dependence low grey level emphasis | HighDependenceLowGrayLevelEmphasis3D | 0.237 | 0.697 | 0.426 | 1.661 |  | 0.757 |
|  | **High dependence high grey level emphasis** | **HighDependenceHighGrayLevelEmphasi3D'** | **56031.250** | **136557.830** | **20700.154** | **22382.100** |  | **0.002** |
|  | Grey level non-uniformity | GrayLevelDependenceNonUniformity3D | 1696.579 | 3115.727 | 1061.177 | 1872.381 |  | 0.695 |
|  | Normalized grey level non-uniformity | NormalizedGrayLevelDependenceNonUniformity3D | 0.035 | 0.024 | 0.041 | 0.023 |  | 0.256 |
|  | Dependence count non-uniformity | DependenceCountNonUniformity3D | 5310.564 | 5630.756 | 3118.159 | 2852.793 |  | 0.132 |
|  | Normalized dependence count non-uniformity | NormalizedDependenceCountNonUniformity3D | 0.194 | 0.151 | 0.169 | 0.061 |  | 0.577 |
|  | Dependence count percentage | DependenceCountPercentage3D | 1.000 | 0.000 | 1.000 | 0.000 |  | NaN |
|  | Grey level variance | GrayLevelDependenceVariance3D | 2011.060 | 8699.890 | 144.407 | 186.055 |  | 0.194 |
|  | Dependence count variance | DependenceCountVariance3D | 6.998 | 6.775 | 6.256 | 5.892 |  | 0.635 |
|  | Dependence count entropy | DependenceCountEntropy3D | 8.182 | 0.556 | 7.743 | 0.473 |  | 0.003 |
|  | Dependence count energy | DependenceCountEnergy3D | 0.005 | 0.001 | 0.006 | 0.002 |  | 0.006 |
|  | **Location features** | | | | | |  |  |
|  | Frontal lobe | Frontal lobe | 1.468 | 2.874 | 0.513 | 1.091 |  | 0.746 |
|  | Limbic lobe | Limbic lobe | 5.634 | 5.932 | 2.039 | 3.333 |  | 0.005 |
|  | **Midbrain** | **Midbrain** | **2.114** | **5.011** | **10.336** | **12.273** |  | **0.001** |
|  | Occipital lobe | Occipital lobe | 0.337 | 0.647 | 0.099 | 0.226 |  | 0.426 |
|  | Parietal lobe | Parietal lobe | 0.169 | 0.567 | 0.280 | 1.024 |  | 0.419 |
|  | **Temporal lobe** | **Temporal lobe** | **3.601** | **4.301** | **0.344** | **1.221** |  | **0.005** |
|  | Sublobar | Sublobar | 4.376 | 9.132 | 4.255 | 5.105 |  | 0.426 |
|  | Insula | Insula | 4.578 | 10.001 | 0.256 | 0.931 |  | 0.106 |
|  | Basal ganglia | Basal ganglia | 5.034 | 13.808 | 5.294 | 8.187 |  | 0.456 |
|  | Thalamus | Thalamus | 5.298 | 16.473 | 6.541 | 10.619 |  | 0.048 |
