# Supplementary figures and images for: Morphometric and radiomics analysis toward the prediction of epilepsy associated with supratentorial low-grade glioma in children
Source: Cancer Imaging. 2025 May 19;25:63. doi: 10.1186/s40644-025-00881-1 (PMC12090388; doi:10.1186/s40644-025-00881-1)

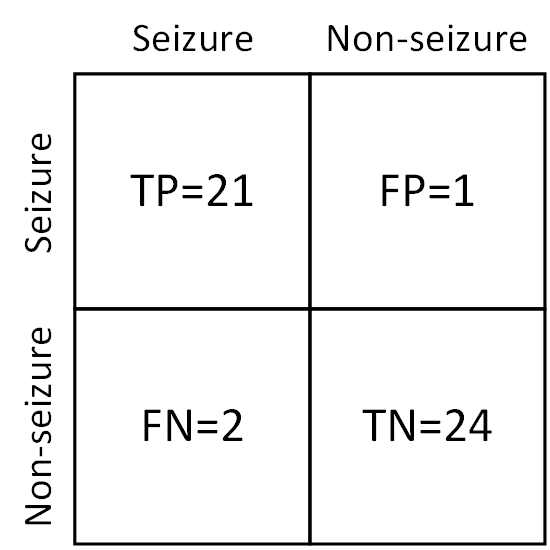

Supplement: Supplementary file 2 — Supplementary Material 2: Supplementary fig. 1: Confusion matrix illustrating the classification performance of the best-performing model. The matrix includes true positives (TP = 21), false negatives (FN = 2), false positives (FP = 1), and true negatives (TN = 24). Positive predictive value (PPV) and negative predictive value (NPV) were calculated as 0.955 and 0.923, respectively, providing additional context for the model’s diagnostic performance. [file 40644_2025_881_MOESM2_ESM.tif]
